# Chronic vagal nerve stimulation has no effect on tachycardia‐induced heart failure progression or excitation–contraction coupling

**DOI:** 10.14814/phy2.14321

**Published:** 2020-01-20

**Authors:** Emma J. Radcliffe, Charles M. Pearman, Amy Watkins, Michael Lawless, Graeme J. Kirkwood, Sophie N. Saxton, David A. Eisner, Andrew W. Trafford

**Affiliations:** ^1^ Unit of Cardiac Physiology Institute of Cardiovascular Sciences Manchester Academic Health Sciences Centre The University of Manchester Manchester UK

**Keywords:** calcium, heart failure, parasympathetic, tachycardia‐induced cardiomyopathy, vagal nerve stimulation

## Abstract

Autonomic dysregulation plays a key role in the development and progression of heart failure (HF). Vagal nerve stimulation (VNS) may be a promising therapeutic approach. However, the outcomes from clinical trials evaluating VNS in HF have been mixed, and the mechanisms underlying this treatment remain poorly understood. Intermittent high‐frequency VNS (pulse width 300 µs, 30 Hz stimulation, 30 s on, and 300 s off) was used in healthy sheep and sheep in which established HF had been induced by 4 weeks rapid ventricular pacing to assess (a) the effects of VNS on intrinsic cardiac vagal tone, (b) whether VNS delays the progression of established HF, and (c) whether high‐frequency VNS affects the regulation of cardiomyocyte calcium handling in health and disease. VNS had no effect on resting heart rate or intrinsic vagal tone in the healthy heart. Although fewer VNS‐treated animals showed subjective signs of heart failure at 6 weeks, overall VNS did not slow the progression of clinical or echocardiographic signs of HF. Chronic VNS did not affect left ventricular cardiomyocyte calcium handling in healthy sheep. Rapid ventricular pacing decreased the L‐type calcium current and calcium transient amplitude, but chronic VNS did not rescue dysfunctional calcium handling. Overall, high‐frequency VNS did not prevent progression of established HF or influence cellular excitation–contraction coupling. However, a different model of HF or selection of different stimulation parameters may have yielded different results. These results highlight the need for greater insight into VNS dosing and parameter selection and a deeper understanding of its physiological effects.

## Graphical abstract

The effects of chronic vagal nerve stimulation (VNS) on cardiac function and calcium cycling were assessed in healthy sheep and sheep in which heart failure had been induced by rapid pacing. VNS did not affect calcium cycling in health or disease states and did not improve echocardiographic or biochemical markers of heart failure.

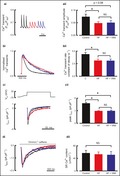

## 1 INTRODUCTION

Heart failure (HF) represents a final common pathway for a wide variety of cardiovascular diseases. Despite advances in the treatment of HF and its underlying causes, mortality remains high (Ponikowski et al., 2016). Autonomic regulation plays an important role in the development and progression of HF. HF is associated with increased sympathetic tone (Cohn et al., 1984), leading to decreased myocardial β‐adrenergic responsiveness (Briston et al., 2011; Chattopadhyay et al., 2010; Horn et al., 2016), and reduced parasympathetic influence which may stem from a loss of transmission at the level of the parasympathetic ganglia (Bibevski & Dunlap, 1999; Eckberg, Drabinsky, & Braunwald, 1971; Horn et al., 2016). The resultant loss of heart rate variability is a marker of poor prognosis in HF (Nolan et al., 1998).

To rescue this autonomic imbalance, sympathetic modulation using β‐adrenoceptor blockade has become a well‐established treatment for HF, improving symptoms and prognosis (Ponikowski et al., 2016). To address the loss of parasympathetic tone, vagal nerve stimulation (VNS) has received increasing interest (Premchand et al., 2014; Schwartz et al., 2008; Zannad et al., 2015). VNS reduced mortality by 36% in a rat myocardial infarction‐induced model of HF (Li et al., 2004), with similar effects reproduced in dogs (Hamann et al., 2013; Zhang et al., 2009). Clinical studies have reported mixed results, with some describing improved ejection fractions and symptoms (Premchand et al., 2014; Schwartz et al., 2008), although others have found no difference in LV systolic function or mortality (Gold et al., 2016; Zannad et al., 2015).

Several potential mechanisms underlying the beneficial effects of VNS have been proposed, including improved hemodynamics, a beneficial inflammatory profile, electrophysiological remodeling, and regulation of nitric oxide signaling (see Radcliffe & Trafford, 2017 for review). Studies using rapid pacing models of HF (Zhang et al., 2009) and human clinical trials using stimulation amplitudes lower than that required to elicit heart rate reductions (Zannad et al., 2015) suggest that decreased heart rate is not required to obtain therapeutic benefit from VNS.

Another potential mechanism by which VNS may improve HF is modulation of excitation–contraction coupling. Dysregulation of calcium cycling is a core component of HF pathophysiology, decreasing sarcoplasmic reticulum calcium content, *I*_Ca‐L_, and calcium transient amplitude (Briston et al., 2011; Hobai & O'Rourke, 2001; Li et al., 2004; Piacentino et al., 2003). This dysregulation not only leads to contractile dysfunction but also contributes to HF‐associated arrhythmias (reviewd by Denham et al., 2018). Calcium handling is influenced by the autonomic nervous system through β‐adrenergic signaling pathways, regulated by acetylcholine (Paterson, 2001), raising the possibility that VNS exerts its influence through alterations in calcium cycling. However, little is known about the effects of chronic VNS in HF on inotropy, β‐adrenergic responsiveness, or whether it restores cardiomyocyte calcium handling that has been perturbed by disease.

Using a translationally relevant large mammalian model employing rapid ventricular pacing to eliminate the role of rate reduction, we hypothesized that chronic intermittent high‐frequency VNS would improve established heart failure through modulation of cardiac excitation–contraction coupling. The aims of this study were as follows: (a) to assess the effect of intermittent VNS on intrinsic cardiac vagal activity in the healthy heart; (b) to assess whether intermittent VNS improved clinical, echocardiographic, and biochemical features of heart failure when LV dysfunction was already established; and 3) to determine whether calcium handling is affected by VNS in healthy and diseased cardiomyocytes.

## 2 METHODS

All procedures received approval from the University of Manchester Animal Welfare and Ethical Review Process and were carried out in accordance with the United Kingdom Animals Scientific Procedures Act (1986), the European Directive 2010/63/EU, and the ARRIVE guidelines (Kilkenny et al., 2010).

### 2.1 Experimental subjects

A total of 48 female Welsh mountain sheep, weighing 31.9 ± 1.3 kg, aged 18 ± 6 months, and treatment‐naïve prior to the start of the study were acquired from The University of Manchester Biological Services Facility. Animals were housed in pens of 3–5 animals with access ad libitum to food and water, and maintained on a 12‐hr light–dark cycle. All experiments were carried out during the working day. Animals in the healthy arm of this study received vagal nerve stimulator implants (*N* = 10) or sham implants only (*N* = 4), to serve as controls for in vivo experiments. Animals in the heart failure arm were randomized prior to surgical instrumentation to either the HF (*N* = 15) or the HF plus VNS (*N* = 10) treatment groups. All animals in this experimental arm underwent cardiac pacemaker implantation, but only animals in the treatment group received vagal nerve stimulator implants. Uninstrumented animals (*N* = 11) were used as controls in patch‐clamp experiments.

### 2.2 Device implantation

An ovine model of HF was selected based on the anatomical and electrophysiological similarities of sheep and human hearts (Hasenfuss, 1998; Lompre et al., 1981). A rapid pacing model, described in detail previously (Briston et al., 2011; Caldwell et al., 2014; Clarke et al., 2014; Horn et al., 2016; Lawless et al., 2019) was used with the specific aim of obviating any heart rate–dependent effects of VNS in HF. Devices were implanted under general anesthesia induced using isoflurane (3%–5% v/v in a 50:50 mix of oxygen and nitrous oxide) and maintained using isoflurane (1%–3% v/v in oxygen). Perioperative analgesia was provided by meloxicam (0.05 mg·kg^−1^) and antibiosis with amoxicillin (15 mg·kg^−1^). Active fixation pacing leads were advanced to the right ventricular apex via a venotomy in the right common jugular vein. Pacing leads were attached via custom made IS‐1 branching connectors (Oscor Inc.) to dual‐chamber cardiac pacemakers (Medtronic, Minnesota, USA) and secured in a subcutaneous pocket in the right cervical region.

In animals allocated to the VNS groups, approximately 2 cm of the right cervical vagus was exposed using blunt dissection. The electrode coils of the VNS lead (LivaNova) were positioned around the nerve with the anode at the cranial and cathode at the caudal end. Care was taken to prevent drying of the exposed nerve section during dissection and electrode placement. Successful placement was confirmed by observing a reduction in heart rate in response to stimulation. The VNS lead was attached to the pulse generator (LivaNova) and the device buried in a subcutaneous pocket a minimum of 20 cm away from the cardiac pacemaker.

### 2.3 Rapid ventricular pacing and VNS protocols

For animals receiving VNS alone, VNS was initiated 7 days following surgery and maintained for 6 weeks. For all VNS animals a manufacturer‐recommended intermittent stimulation protocol was used: pulse width 300 µs, 30 Hz stimulation, 30 s on, and 300 s off. Stimulation amplitude was adjusted biweekly to achieve a 20%–30% reduction in resting heart rate during VNS “on” periods.

For animals in the heart failure arm, rapid ventricular pacing at 210 beats per minute began 1 week after surgery. In the VNS cohort, VNS was initiated 28 days into the rapid pacing protocol and delivered concomitantly with right ventricular tachypacing. Rapid ventricular pacing ± VNS was delivered until animals reached the predetermined end point of 7 weeks rapid ventricular pacing, or when animals displayed subjective signs of HF defined as including lethargy, tachypnea, or the presence of edema upon clinical examination. Animals in either cohort who reached this end point prior to 28 days rapid pacing (i.e., before activation of VNS) were excluded from in vivo analysis. Animals reaching this end point were euthanized using intravenous pentobarbitone (200 mg/kg) mixed with heparin (10,000 IU).

### 2.4 In vivo outcome measures

Rapid pacing and VNS were deactivated for approximately 10 min prior to echocardiography and electrocardiography. All measures were taken from conscious, nonsedated, gently restrained animals held in a seated position. Rapid pacing and VNS were reinitiated immediately upon completion of in vivo assessments.

Right parasternal transthoracic echocardiography (Vivid 7; GE Healthcare) was used to assess cardiac dimensions and function. Assessment of LV systolic function was performed using short‐axis fractional area change and M‐mode fractional shortening at a level immediately apical to the papillary muscles. Ejection fraction was not calculated as, due to the anatomical alignment of the ventricular apex over the sternum in sheep, it was not possible to reliably obtain four‐chamber long‐axis views. A five‐lead ECG (Iox, EMKA Technologies) was used to determine heart rate and PR interval from ECGs digitized at 1 kHz as previously described (Horn et al., 2016). Frequency domain heart rate variability (HRV) was assessed by discrete Fourier transformation of RR interval data. The bandwidths selected for high‐frequency (HF) and low‐frequency (LF) HRV were 0.15–1.5Hz and 0.04–0.15 Hz, respectively. Cardiac vagal influence was assessed as described previously (Horn et al., 2016) by assessing heart rate changes in response to sequential pharmacological autonomic blockade. Sympathetic blockade was achieved using a single intravenous administration of propranolol (0.5 mg·kg^−1^, prepared as propranolol hydrochloride in 0.9% NaCl solution, Sigma‐Aldrich). Full autonomic blockade was achieved approximately 5 min later by blocking parasympathetic influence with an intravenous bolus of atropine (0.05 mg·kg^−1^, prepared as atropine sulfate in 0.9% NaCl solution; Sigma‐Aldrich) and 0.05 mg·kg^−1^·min^−1^ atropine infusion until autonomic assessment was complete.

### 2.5 qPCR

Sections of left ventricular posterior free wall were snap frozen in liquid nitrogen immediately upon euthanasia and assayed by quantitative real‐time PCR (qPCR) analysis of B‐type natriuretic peptide (NBBP) mRNA levels, as previously described (Lawless et al., 2019). RNA was extracted by homogenizing tissue in TRIzol and phases were separated with cholorform. Subsequently, the aqueous phase was purified using RNeasy Fibrous Tissue Mini Kit (Qiagen) following the manufacturers' recommendations. RNA yield and integrity was assessed using a Nanodrop (Thermo Fisher Scientific) and Tapestation (Agilent). Only RNA obtaining RIN scores of >7.5 were used during this study. cDNA was generated using a high‐capacity reverse transcription RNA to cDNA kit (Applied Biosystems) as per the manufacturers’ recommendation. qPCR was performed using TaqMan FAM‐MGB assays with probes designed specifically to ovine gene variants (RPLP0‐Oa04824509, NBBP‐Oa04931155, both from Thermo Fisher Scientific), following the manufacturers' protocol. Data were normalized to the housekeeper RPLP0 using the 2^−∆∆Ct^ method (Lawless et al., 2019). Data are presented as the average of three technical replicates.

### 2.6 Voltage clamp studies

Midmyocardial myocytes were enzymatically isolated from the anterior wall of the left ventricle for whole‐cell voltage clamp experiments, as previously described (Briston et al., 2011; Caldwell et al., 2014; Lawless et al., 2019). Cells were bathed in experimental solution containing ([in mmol/l] BaCl_2_, 0.1; CaCl_2_, 1.8; DIDS, 0.1; Glucose, 10; HEPES, 10; KCl, 4; MgCl_2_, 1; NaCl, 140; Probenicid, 2; 4‐aminipyridine, 5; pH to 7.34 with NaOH). Electrodes (2–3 MΩ) were filled with pipette solution containing (in mmol/l) CaCl_2_, 0.28; CsCl, 118; CsEGTA, 0.02; HEPES, 10; MgCl_2_, 4; Na_2_ATP, 3.1; Na_2_GTP, 0.42; Phosphocreatine, 3; pH 7.2 with CsOH. Cells were stimulated at a rate of 0.5Hz using a ramped protocol; from a holding potential of −60 mV, membrane potential was ramped to −40 mV before a 50 ms duration step to 10 mV using an Axopatch 700A and pCLAMP software. Series resistance compensation (80%–90%) was applied to minimize voltage errors. Intracellular calcium levels were measured throughout by pipette loading cells using Fura‐2, pentapotassium salt (100 µmol/l; Thermo Fisher Scientific). Florescence was excited using an Optosource light and Optoscan monochrometer (both Cairn Research) to rapidly alternate between the excitation wavelengths 340 and 380 nm. A ratio of emission at the two wavelengths (R_340/380_) was used to determine intracellular calcium levels. SR calcium content was calculated as previously described (Dibb, Rueckschloss, Eisner, Isenberg, & Trafford, 2004; Varro, Negretti, Hester & Eisner, 1993) by rapidly applying caffeine (10 mmol/l) and integrating the resultant inward sodium–calcium exchanger current.

### 2.7 Statistics

Data are presented as mean ± the standard error of the mean (*SEM*) for n observations in *N* animals. Where multiple observations (*n*) were made from the same animal (N), linear mixed‐model analysis (SPSS Statistics, IBM) was used to account for the nested design of the experiment, as described previously (Caldwell et al., 2014; Clarke et al., 2017; Lawless et al., 2019; Pearman et al., 2018). Survival curves were assessed using GraphPad Prism survival curve analysis (GraphPad Software Inc.). Other variables were assessed using *t* tests or repeated measures ANOVA for continuous data and Chi‐square or Fisher's exact test for categorical data where appropriate (SigmaPlot, Systat Software). Nonnormally distributed data were log_10_ transformed prior to statistical analysis. Values of *p* < .05 were considered significant.

## 3 RESULTS

### 3.1 VNS does not affect intrinsic cardiac vagal tone

**Figure 1:**
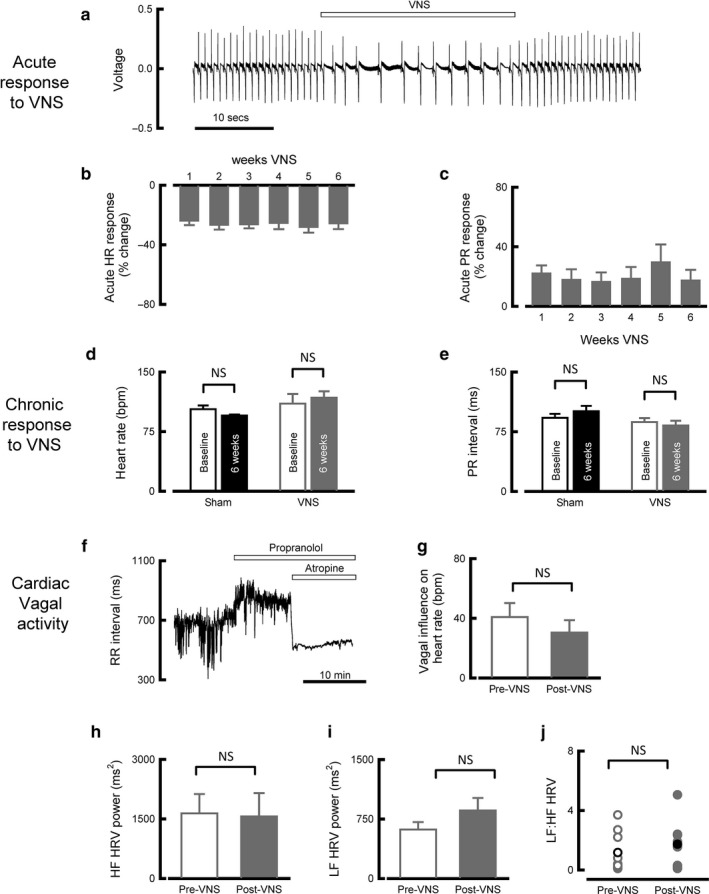
Chronic VNS treatment does not induce long‐term upregulation of cardiac vagal activity. (a) Representative surface ECG showing the acute response to a bout of VNS. (b) Summary data showing the acute bradycardic response to intermittent VNS over a 6‐week time course. (*N* = 8). (c) Summary data showing acute lengthening of the PR interval in response to intermittent VNS over a 6‐week time course. (*N* = 8). (d) Summary data showing sinus rhythm in sham and VNS animals. (*N* = sham 4, VNS 9). (e) Summary data showing PR interval in sham and VNS animals. (*N* = sham 4, VNS 9). (f) Representative tachogram recorded during pharmacological autonomic blockade using 0.5 mg/kg propranolol and 0.05 mg/kg atropine. (g) Paired summary data showing the vagal influence on heart rate as assessed using pharmacological autonomic blockade (*N* = 10). (h) Paired summary data showing the power of HF HRV (*N* = 9). (i) Paired summary data showing the power of LF HRV (*N* = 9). (j) Paired summary data showing the LF:HF HRV (black indicates mean values, *N* = 9). Open bars indicate pre‐ and closed bars indicate post‐VNS or sham stimulation. NS *p* > .05

We first assessed the feasibility of intermittent vagal nerve stimulation in healthy sheep. Animals tolerated VNS at amplitudes required to produce an acute heart rate reduction of ~20%, similar to that achieved with β‐blocker therapy (McAlister, Wiebe, Ezekowitz, Leung, & Armstrong, 2009) (Figure 1a). Stimulation amplitude was restricted by the development of a VNS‐dependent cough in one animal; in this case, stimulation amplitude was gradually up‐titrated over a 1‐week period to achieve the desired degree of heart rate reduction along with cough desensitization. Bradycardia and PR interval prolongation were consistently observed during the ‘on’ periods of stimulation over the 6‐week time course (Figure 1b and c, *N* = 8).

We next explored the effect of chronic VNS on intrinsic cardiac vagal tone. Following 6 weeks intermittent VNS, resting heart rate and PR interval during ‘off’ periods were similar to baseline measurements recorded prior to initiation of VNS (heart rate: baseline 110 ± 12/min, post‐VNS 118 ± 8/min, *N* = 9, *p* = .29, Figure 1d; PR interval: baseline 87 ± 5 ms, post‐VNS 83 ± 6 *N* = 9, *p* = .10, Figure 1e). We investigated this further using pharmacological autonomic blockade to sequentially block sympathetic activity with propranolol and parasympathetic activity with atropine, then quantifying vagal influence as the increase in heart rate following atropine administration (Figure 1f). Vagal tone was similar to baseline following 6 weeks VNS (baseline + 41 ± 9/min, post‐VNS + 30 ± 8/min, *N* = 10, *p* = .19, Figure 1g). Frequency domain HRV was used as a secondary means of quantifying cardiac autonomic balance. No changes in high‐frequency HRV were seen following 6 weeks VNS (baseline 1,640 ± 458 ms^2^, post‐VNS 1,562 ± 554 ms^2^, *N* = 9, *p* = .87, Figure 1h), low‐frequency HRV (baseline 617 ± 89 ms^2^, post‐VNS 859 ± 148 ms^2^, *N* = 9, *p* = .21, Figure 1i), or the ratio of low‐frequency to high‐frequency HRV (baseline 1.17 ± 0.42, post‐VNS 1.73 ± 0.48, *N* = 9, *p* = .28, Figure 1j). Overall, intrinsic cardiac vagal tone was not affected by 6 weeks intermittent VNS.

### 3.2 VNS does not prevent signs of heart failure developing in animals with established ventricular systolic dysfunction

**Figure 2:**
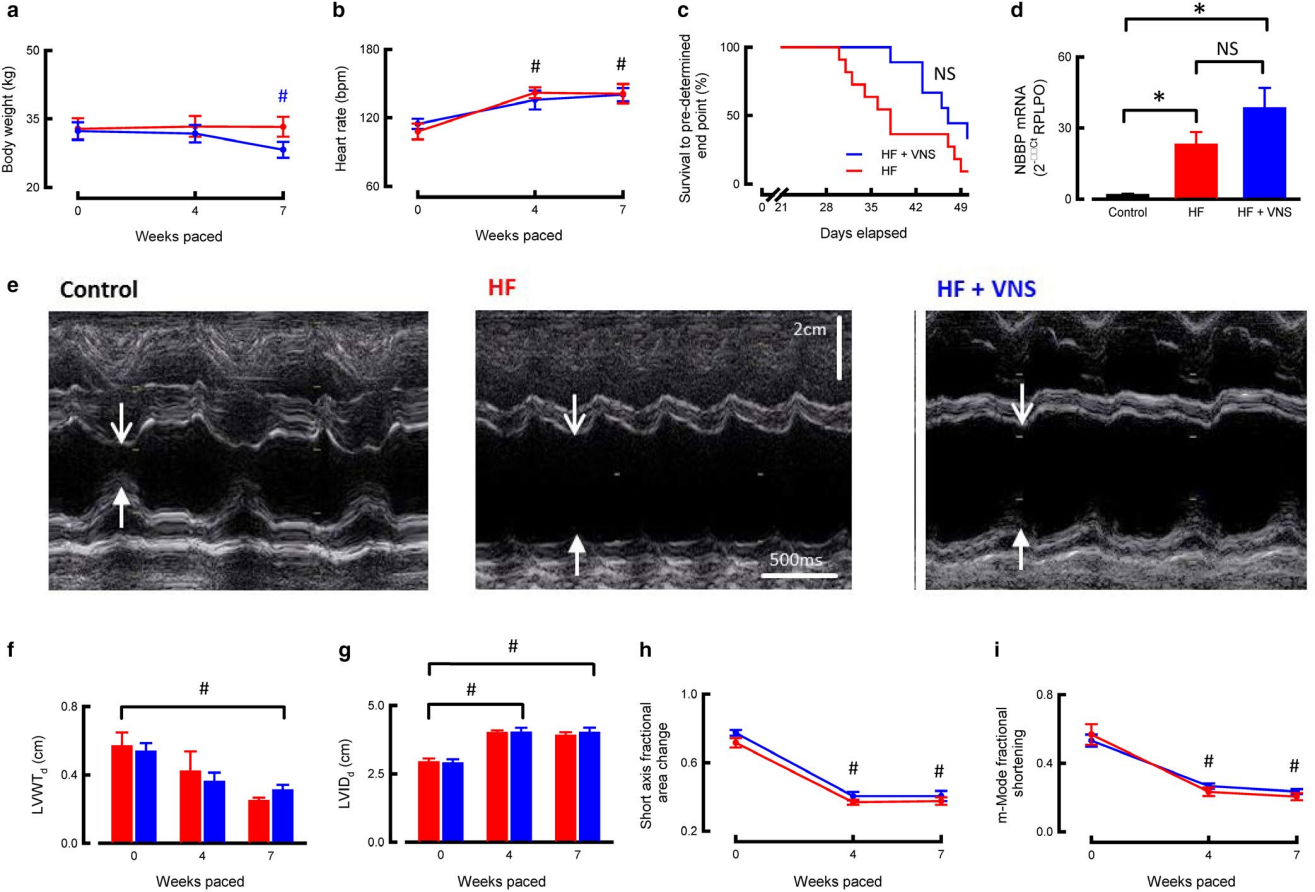
In vivo assessment of VNS treatment in tachypacing‐induced heart failure. (a) Body weight (*N* = HF 8, HF + VNS 8). (b) Resting heart rate (*N* = HF 6, HF + VNS 7). (c) Survival plot showing the percentage of animals free from signs of heart failure (*N* = HF 11, HF + VNS 9). VNS treatment commenced after 28 days pacing (dotted line). (d) Left ventricular NBBP mRNA levels (*N* = control 8, HF 8, HF + VNS 8). (e) Representative m‐mode echocardiographic images from control (left), HF (center), and HF + VNS (right). Open arrows indicate septal wall and closed arrows indicate left ventricular free wall. (f) Summary data of echocardiographic assessment showing left ventricular free wall thickness in diastole (LVWT_d_)( *N* = HF 7–10, HF + VNS 7–8). (g) Summary data of echocardiographic assessment showing left ventricular internal diameter in diastole (LVIDd) (*N* = 8–10, HF + VNS 7–9). (h) Summary data of echocardiographic assessment showing m‐mode fractional shortening (*N* = HF 8–10, HF + VNS 7–9). (i) Summary data of echocardiographic assessment showing short‐axis fractional area change (*N* = HF 10, HF + VNS 7–9). ^NS^ *p* > 0.05, *p < .05 between groups, ^#^ *p* < .05 versus baseline, data all presented as mean ± *SEM*

Having determined that intermittent chronic VNS was safe and that consistent heart rate reductions were achievable during the VNS “on” periods, we next investigated the effect of VNS in animals with established heart failure. Animals assigned to treatment with VNS had similar body weight, resting heart rate, and echocardiographic measures of left ventricular structure and function compared to those assigned to the nontreatment group (Figure 2a–i). One animal assigned to the VNS study arm and four animals assigned to the untreated study arm exhibited signs of end‐stage HF prior to day 28 of the protocol (i.e., prior to any potential delivery of VNS therapy) and were thus excluded from further analysis.

Four weeks rapid ventricular pacing caused LV systolic dysfunction as evidenced by decreased fractional shortening, fractional area change, and LV posterior wall thickness in diastole (LVWTd), and increased LV internal diameter in diastole (LVIDd) (Figure 2e–i). Although at week 6, fewer animals in the VNS‐treated cohort had shown signs of HF (HF 8/9 [89%], VNS 4/11 [36%], *p* = .03), this effect was not sustained and overall VNS did not significantly delay development of signs of HF (HF 39.7 days, 95% CI 34.8–44.6 days, VNS 46.2 days, 95% CI 43.4–49.2 days, *p* = .12 by LogRank, Figure 2c). Clinical signs of end‐stage heart failure were seen in similar proportions of VNS‐treated and untreated animals by 7 weeks (HF 10/11 [91%]), VNS 7/9 [78%], *p* = .59).

This progression of heart failure beyond 4 weeks was not reflected in any further deterioration in echocardiographic variables. At the end of the rapid pacing protocol (7 weeks or end‐stage HF) no differences were seen between VNS‐treated and untreated groups in LVWTd (25 ± 2 mm in untreated and 31 ± 3 mm in treated, *p* = .13, Figure 2f), LVIDd (39.0 ± 12 mm in untreated and 40.0 ± 19 mm in treated, *p* = .67, Figure 2g), fractional area change (0.38 ± 0.02 in untreated and 0.40 ± 0.02 in treated, *p* = .43, Figure 2h), or m‐mode fractional shortening (0.20 ± 0.02 in untreated and 0.23 ± 0.01 in treated, *p* = .21, Figure 2i).

Animals undergoing rapid ventricular pacing lost weight during the development of HF (baseline 32.3 ± 1.8 kg, end‐stage HF 28.2 ± 1.6 kg, *N* = 8 in each group, *p* < .01, Figure 2a), but weight was similar between treated and untreated animals at all timepoints (*p* = .43, Figure 2a). Resting heart rate (measured during discontinuation of pacing) was increased in all animals at the 4‐week timepoint (baseline 111.6 ± 3.9/min, 4 weeks 138.5 ± 4.9/min, *p* < .01, Figure 2b) and remained elevated throughout the remainder of the protocol for both treated and untreated animals (end‐stage treated 140.2 ± 5.8/min, untreated 141.1 ± 8.5/min, *p* = .94, Figure 2b). Left ventricular B‐type natriuretic peptide (NBBP) mRNA levels were higher in tissue from animals with untreated HF than controls (normalized 2^−ΔΔCt^: control 1.6 ± 0.5, HF 22.9 ± 5.3, *N* = 8 in each group, *p* < .01, Figure 2d), but did not differ between VNS‐treated and untreated animals (treated 38.3 ± 8.6, untreated 22.9 ± 5.3, *N* = 8 in each group, *p* = .15, Figure 2d).

Overall, VNS did not prevent development of subjective signs of end‐stage HF in sheep with established LV systolic dysfunction, or did it affect HF‐induced weight loss, tachycardia, or NBBP mRNA measured at end‐stage HF.

### 3.3 VNS does not affect ventricular myocyte calcium handling in health or disease

**Figure 3:**
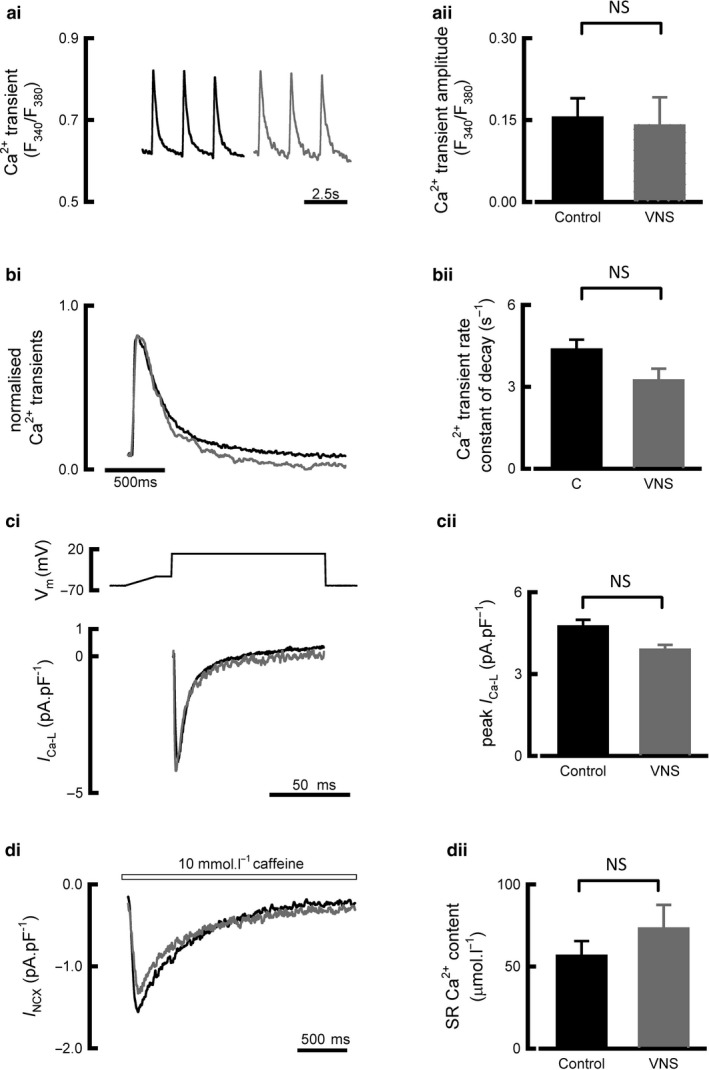
Normal intracellular Ca^2+^ handing is unaffected by 6 weeks VNS. (ai) Representative Ca^2+^ florescent recordings from voltage clamp, stimulated cells. (aii) Summary data for Ca^2+^ transient amplitude (*N* hearts/*n* cells = control 10/42, VNS 3/14). (bi) Normalized and superimposed Ca^2+^ transients. (bii) Summary data for Ca^2+^ transient rate constant of decay (N hearts/n cells = control 10/42, VNS 3/14). (ci) Representative I_ca‐L_ current recordings normalized to cellular capacitance. (cii) Summary data for peak I_Ca‐L_ (*N* hearts/*n* cells = control 18/55, VNS 6/24). (di) Representative caffeine‐induced NCX currents. (dii) Summary data for SR Ca^2+^ content (*N* hearts/*n* cells = control 6/18, VNS 3/6). ^NS^ *p* > 0.05, data all presented as mean ± *SEM*

We next explored whether 6 weeks VNS influenced calcium handling in cardiomyocytes from healthy sheep. VNS had no effect on calcium transient amplitude (R_340/380_ in control 0.15 ± 0.03, *N* = 10, *n* = 42; VNS 0.14 ± 0.05, *N* = 3, *n* = 14; *p* = .84; Figure 3a) or the rate constant of decay of the calcium transient (control 4.36 ± 0.36/s; VNS 3.23 ± 0.42/s, *p* = .14, Figure 3b). Factors associated with the regulation of calcium transient amplitude were also investigated; no difference in peak *I*_Ca‐L_ (control 4.74 ± 0.24 pA/pF, *N* = 18, *n* = 55; VNS 3.89 ± 0.18 pA/pF, *N* = 6, *n* = 24; *p* = .22, Figure 3c) or SR calcium content (control 56.5 ± 8.8 µmol/l, *N* = 6, *n* = 18; VNS 73.0 ± 13 µmol/l, *N* = 3, *n* = 6, *p* = .36, Figure 3d) was observed between ventricular myocytes isolated from VNS or control animals.

**Figure 4:**
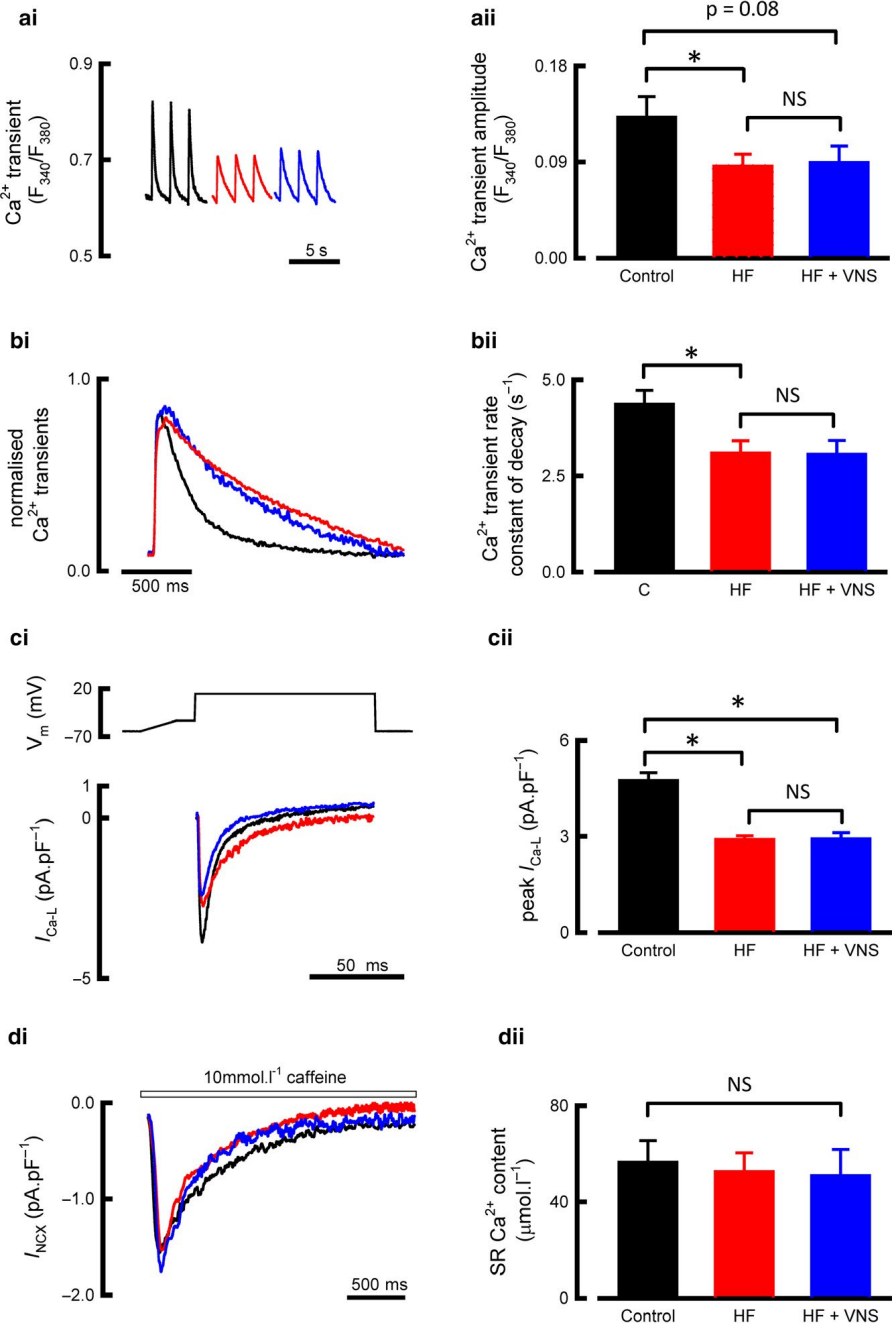
HF‐induced intracellular Ca^2+^ handling dysfunction is unaffected by VNS treatment. (ai) Representative Ca^2+^ fluorescent recordings from voltage clamp, stimulated cells. (aii) Summary data for Ca^2+^ transient amplitude (*N* hearts/*n* cells = control 8/35, HF 9/34, HF + VNS 5/17). (bi) Normalized and superimposed Ca^2+^ transients. (bii) Summary data for Ca^2+^ transient rate constant of decay (N hearts/n cells = control 10/42, HF 10/44, HF + VNS 5/19). (ci) Representative I_ca‐L_ current recordings normalized to cellular capacitance. (cii) Summary data for peak I_Ca‐L_ (*N* hearts/*n* cells = control 18/55, HF 8/31, HF + VNS 6/19). (di) Representative caffeine‐induced NXC currents. (dii) Summary data for SR Ca^2+^ content (*N* hearts/*n* cells = control 6/18, HF 7/15, HF + VNS 3/8). ^NS^ *p* > .05, *p < .05 between groups, all presented as mean ± *SEM*

We continued by examining whether VNS rescued the dysfunctional calcium handling associated with HF. HF was associated with a 44% decrease in calcium transient amplitude (R_340/380_ in control 0.13 ± 0.02, *N* = 8, *n* = 35; HF 0.09 ± 0.01, *N* = 9, *n* = 34; *p* = .02, Figure 4a), and no change in the rate constant of decay of the calcium transient (control 4.36 ± 0.36/s, *N* = 10, *n* = 42; HF 3.09 ± 0.31/s, *N* = 10, *n* = 44; *p* = .19; Figure 4b). HF was also associated with decreased *I*_Ca‐L_ (control 4.7 ± 0.2 pA/pF, *N* = 18, *n* = 55; HF 2.9 ± 0.1 pA/pF, *N* = 8, *n* = 31, *p* = .01, Figure 4c).

VNS did not rescue this dysfunctional pattern of calcium handling. No differences between treated and untreated groups was seen in calcium transient amplitude (R_340/380_ in HF 0.09 ± 0.01; *N* = 9, *n* = 34; VNS 0.09 ± 0.01, *N* = 5, *n* = 17, *p* = .43, Figure 4a), rate constant of decay of the calcium transient (HF 3.09 ± 0.31/s, *N* = 10, *n* = 44; VNS 3.07 ± 0.31/s, *N* = 5, *n* = 19; *p* = .69, Figure 4b), or peak *I*_Ca‐L_ (HF 2.87 ± 0.12 pA/pF, *N* = 8, *n* = 31; VNS 2.92 ± 0.19 pA/pF, *N* = 6, *n* = 19; *p* = .07; Figure 4c). SR calcium content remained unchanged across the three groups (control 56.5 ± 8.8 µmol/l, *N* = 6, *n* = 18; HF 64.2 ± 16.1 µmol/l; *N* = 7, *n* = 15; VNS 50.8 ± 10.3 µmol/l, *N* = 3, *n* = 8; *p* = .99, Figure 4d). Overall, while HF was associated with dysfunctional cardiomyocyte calcium handling, VNS did not affect calcium handling in health or rescue the dysfunction associated with HF in animals displaying signs of end‐stage HF.

## 4 DISCUSSION

The main findings of this study are as follows: first, in healthy sheep, 7 weeks of intermittent high‐frequency VNS did not affect intrinsic cardiac autonomic balance. Second, VNS neither prevented development of subjective signs nor reversed echocardiographic features of heart failure in sheep with established LV dysfunction induced by rapid ventricular pacing. Third, VNS did not alter the calcium handling properties of cardiomyocytes from healthy sheep and did not attenuate or reverse dysfunctional calcium handling in cardiomyocytes from sheep with heart failure. This study is the first to demonstrate that cellular calcium is not influenced by intermittent chronic high‐frequency VNS and echoes the lack of therapeutic benefit found with this treatment in some recent clinical trials.

### 4.1 VNS as a means of altering cardiac autonomic balance

In healthy animals, VNS slowed heart rate during the 30 s “on” periods, but during the 300 s “off” periods heart rate returned to baseline with no signs of increased intrinsic vagal tone measured by HRV or pharmacological autonomic blockade.

There is some empirical evidence that chronic VNS influences intrinsic cardiac vagal tone, derived from observations that following a sustained period of intermittent VNS a sustained heart rate reduction is seen during VNS ‘off’ periods of greater magnitude than the acute rate reduction seen during VNS ‘on’ periods. However, results have been mixed, ranging from a modest reduction to no effect (De Ferrari et al., 2011; Li et al., 2004; Hamann et al., 2013; Premchand et al., 2014; Schwartz et al., 2008; Zhang et al., 2009). Furthermore, a single study suggested that VNS persistently increased intrinsic vagal tone as measured by baroreflex sensitivity and HRV (Zhang et al., 2009). These heterogeneous results may reflect differences in stimulation protocols and the complex interplay among VNS, central, and peripheral neuronal circuits (Ardell et al., 2017).

The mechanisms underpinning this effect remain unclear. The neural control of heart rate is complex, comprising a multilayered network of intrinsic cardiac neurons modulated by the autonomic nervous system and by cardiac mechanoreceptors (Beaumont et al., 2013). This network has great scope for plasticity in response to chronic VNS, although how this might occur is yet to be elucidated. As the reduction in vagal tone seen in heart failure has been associated with a loss of nicotinic acetylcholine receptors on the postsynaptic surface (Bibevski & Dunlap, 2011), it is possible that the restoration of vagal tone seen in some models of VNS may be caused by restoration of these receptors. However, to the best of our knowledge this has not been investigated.

At the cervical level, the vagus nerve is predominantly sensory, comprising approximately 80% afferent and 20% efferent fibers. The activation threshold for afferent fibers is lower than efferent, and at lower stimulation amplitudes, frequencies, and pulse widths, afferent fibers are predominantly activated. This preferential afferent activation suppresses central parasympathetic outflow leading to an increase in heart rate (Ardell et al., 2017; Ardell, Rajendran, Nier, KenKnight, & Armour, 2015). With more aggressive stimulation, the effects of efferent activation outweigh afferent activation resulting in the classic bradycardic response to VNS. These parameters can be manipulated to elicit equal activation of central and peripheral vagal circuits thereby causing no effect on heart rate, a point referred to as the neural fulcrum (Ardell et al., 2017). The manufacturer‐recommended high stimulation frequency (30 Hz), and relatively high stimulation amplitudes needed to cause a 20% heart rate reduction used here (1.18 ± 0.12 mA) demonstrated strong efferent activation. This degree of activation is likely to have suppressed central parasympathetic outflow and may have been sufficient to elicit a reflex sympathetic response which could overwhelm the increase in cardiac vagal activity. This hypothesis is supported by the trend toward an increase in resting heart rate during VNS ‘off’ periods. If less aggressive VNS had been used in this work, a different effect on intrinsic vagal tone and potentially HF outcomes may have been observed.

### 4.2 VNS as a therapy for HF

In this study, rapid ventricular pacing led to comparable LV systolic dysfunction and a similar increase in left ventricular NBBP mRNA between VNS‐treated and untreated animals. Furthermore, VNS did not prevent the development of subjective signs of HF. These results differ from previous studies of VNS in animal models of HF which found that VNS led to an improvement in markers of heart failure (Hamann et al., 2013; Zhang et al., 2009), reviewed in Radcliffe and Trafford (2017). One explanation for this could be due to differences in experimental design. In this work we initiated VNS after four weeks of rapid pacing in order to ensure that significant cardiac dysfunction was present prior to the onset of treatment, mirroring the use of VNS in clinical settings. However, unlike in clinical use in which the major insult causing HF such as a myocardial infarction occurs as a one off, in this work a potent driver for progression of HF in the form of rapid ventricular pacing continued to be applied during VNS. In contrast, previous work reporting beneficial effects of VNS initiated treatment before LV dysfunction occurred (Zhang et al., 2009), or used postmyocardial infarction models in which a strong driver for ongoing progression of HF was not present (Li et al., 2004; Hamann et al., 2013). The ongoing driver of LV dysfunction used here appeared to ultimately overwhelm any potential benefit from VNS. While this design was suitable to look for a large effect from VNS in delaying the signs of end‐stage heart failure, it may have been insensitive to more modest benefits. Indeed, the echocardiographic measures of LV dysfunction were established by 4 weeks and did not deteriorate further after this. Even if VNS had caused a modest slowing of the progression of HF, this would be unlikely to be reflected in differences in echocardiography unless the effect was stronger than the continued driver of HF progression. Furthermore, as the vast majority of experimental subjects reached end‐stage HF eventually, the similar echocardiographic and biochemical markers of HF between groups measured at this point may merely reflect equivalent degrees of sickness between groups once end stage had been reached.

Another potential explanation for a lack of benefit seen here may be the stimulation frequency used. We used manufacturer‐recommended VNS settings. Clinical trials of VNS have reported variable results. NECTAR‐HF did not find a beneficial effect from VNS, attributing this to the use of high‐frequency (20 Hz) low‐amplitude stimulation, similar to the 30 Hz stimulation used in this study. In contrast, ANTHEM‐HF, using 10 Hz stimulation, demonstrated improved cardiac function and reduced HF symptoms (Premchand et al., 2014). The importance of stimulation frequency relates to the neural fulcrum theory described above, as lower stimulation frequencies may simultaneously activate both central and peripheral vagal circuits and prevent reflex suppression of the other. Stimulating around the neural fulcrum in this way is accompanied by <5% heart rate alteration during the VNS “on” period (Ardell et al., 2017). The VNS stimulation parameters used here and in previous work are outlined in Table 1.

**Table 1:** Summary of the VNS stimulation parameters used compared to the neural fulcrum of a control dog (see row 1)

Study	Species model	Frequency (Hz)	Pulse width (ms)	Amplitude (mA)	Duty cycle	HR reduction	Outcome with VNS
“Neural Fulcrum”	Dog	8–10	0.5	2–2.5		~5%	–
Ardell et al. (2015, 2017)	Healthy control				Untested		
Radcliffe and Trafford (2017)	Sheep	30	0.5	0.75–2	30 s on	~20%	No delay in HF progression
	Rapid pacing				300 s off		No change in LVEF
					(~9%)		
Li et al. (2004)	Rat	20	0.2	0.1–0.3	10 s on	20–30 bpm	↑ survival
	Myocardial infarction				50 s off		↑ LVEF
					(~ 17%)		
Zhang et al. (2009)	Dog	20	0.5	0.75–2.5	14 s on	20 bpm	↑LVEF
	Rapid pacing				12 s off		
					(~54%)		
Schwartz et al. (2008)	Human	–	1.0	1.1–5.5	2–10 s on	5–10 bpm	↓ symptoms
De Ferrari et al. (2011)					6–30 s off		↑ LVEF
					(25%)		
Premchand et al. (2014) (ANTHEM‐HF)	Human	10	0.13	1.4–2.6	14 s on	4–6 bpm	↑ LVEF
					66 s off		
					(~18%)		
Zannad et al. (2015) (NECTAR‐HF)	Human	20	0.3	0.5–1.98	10 s on	–	↓ symptoms
					50 s off		No change in LVEF
					(~17%)		
Gold et al. (2016) (INNOVATE‐HF)	Human	Synchronized ventricular depolarization	0.5	3.9 ± 1.0	Synchronized ventricular depolarization	–	No change in LVEF

Note

Radcliffe and Trafford (2017) outlines the stimulation parameters used in this study.

Abbreviations: HR, heart rate; VNS, vagal nerve stimulation; LVEF, left ventricular ejection fraction.

A third potential explanation is that the lack of benefit in this study could be due to the absence of VNS‐induced bradycardia due to the rapid ventricular pacing used to induce HF. While rate reduction improves hemodynamics which may be an important mediator of the therapeutic effects of β‐blocker therapy (Thackray et al., 2006), heart rate–independent effects of VNS have been demonstrated in previous work (Zhang et al., 2009).

Overall, although no significant slowing of HF progression was demonstrated here, it is possible that a modest benefit from VNS might have been seen if treatment was initiated earlier, if a less aggressive ongoing driver of HF was present, if lower stimulation frequencies closer to the neural fulcrum were used, or if the bradycardic effects of VNS had not been masked.

### 4.3 Excitation–contraction coupling as a potential therapeutic mechanism

As shown previously in this model and by others, HF is associated with a reduction in calcium transient amplitude (Briston et al., 2011; Hobai & O'Rourke, 2001). In this model, the reduction can be explained by decreased *I*_Ca‐L_, the trigger for SR calcium release (Briston et al., 2011). Chronic VNS in healthy animals did not affect calcium handling, reaffirming the safety of this therapy. This study is the first to show that chronic VNS does not reverse the decrease in *I*_Ca‐L_ and calcium transient amplitude in the diseased heart. However, as the majority of animals in both treated and untreated groups had reached a similar degree of HF by the time cardiomyocytes were harvested, a modest beneficial effect of VNS delaying the progression of HF mediated by improvements in calcium cycling may have been missed. If cardiomyocytes had been harvested earlier in the disease process, such as the 6‐week timepoint at which fewer VNS‐treated animals had developed signs of end‐stage HF, differences in calcium cycling may have been revealed.

## 5 LIMITATIONS

The assessment of HF progression was subjective. Furthermore, although animals were randomized to treatment or nontreatment groups prior to surgery and the initiation of rapid pacing, operators were not blinded to treatment allocation. This had the potential to bias the subjective assessment of HF progression. Objective measures of HF progression were limited: echocardiographic measures did not deteriorate further after the 4‐week timepoint at which VNS was initiated, and we were only able to assess BNNP from tissue obtained at end stage, as attempts to measure serum NT‐proBNP were unsuccessful in this species.

Although a significant difference in the progression of HF was not found, the relatively low numbers of experimental subjects limited the statistical power. A larger study may have revealed a modest beneficial effect that this work was not powered to detect.

The absence of a difference in cellular calcium handling may be due to the end point at which cells were isolated. Isolating cells earlier in the disease process may have been more likely to detect a difference in calcium cycling if a modest slowing of disease progression had been present.

## 6 CONCLUSIONS

We have shown that chronic intermittent high‐frequency, high‐amplitude VNS has no long‐lasting effects on cardiac autonomic tone and does not significantly slow the progression of tachycardia‐induced heart failure in sheep once LV dysfunction has already become established. VNS applied in this manner had no effects on ventricular myocyte calcium handling in health or disease. However, a modest effect of VNS on the progression of HF may be more apparent in models in which less potent ongoing drivers of HF progression are used, and it cannot be excluded that in these models an effect on cellular calcium handling may be apparent. We speculate that VNS applied using lower stimulation frequencies closer to the point of the neural fulcrum may be more effective.

## CONFLICT OF INTEREST

The authors declare no competing interests.

## AUTHOR CONTRIBUTIONS

Study concept and design: AWT; supervision: AWT and DAE; animal models, in vivo data generation and analysis: EJR, AWT, CMP, AEW, ML, SNS, and GJK; cell isolation, patch‐clamp experiment and analysis: EJR and ML. The manuscript was written by EJR, CMP, and AWT and reviewed by all authors.

## Data Availability

Data are available upon reasonable request from the authors.
